# A Clinically Ultrasound Fusion Visual Prediction Model for Childhood Irreducible Small-Bowel Intussusception

**DOI:** 10.1007/s12098-026-06147-3

**Published:** 2026-04-29

**Authors:** Hongjia Qiang, Xin Li, Zhaozheng Ding, Xiangjie Li, Yuan Cao, Dongsheng Zhu

**Affiliations:** Department of Pediatric Surgery, The First People’s Hospital of Lianyungang, Affiliated to Kangda College of Nanjing Medical University, Lianyungang, 222000 Jiangsu Province China

**Keywords:** Intussusception, Ultrasound, Small-bowel, Irreducible, Prediction model

## Abstract

Small-bowel intussusception (SBI) is usually transient, but 10% require surgery. The authors developed a bedside nomogram using routine ultrasound to predict irreducible SBI in 484 children (47 irreducible, 9.7%). LASSO regression selected five predictors: bloody stool, intussusception diameter and length, bowel wall thickness, and Doppler flow grade. The nomogram demonstrated good discrimination (C-index 0.907, 95% CI 0.869–0.945) and favorable calibration. This bedside tool enables immediate risk stratification using routine ultrasound measurements, potentially reducing unnecessary surgical delays.

## Introduction

Intussusception is one of the most common acute abdominal conditions in infants and young children [1]. Approximately 90% of pediatric intussusception cases are ileocolic, while small bowel intussusception (SBI) accounts for only 5%–10% [2]. Due to its deep location, air or hydrostatic enema often fails to reach SBI [3]. International consensus guidelines list SBI as a contraindication for enema reduction [4]. Most SBI cases reduce spontaneously within hours to days without intervention [3, 5], but approximately 10% progress to irreducible SBI, which can lead to bowel necrosis without timely recognition. Early identification of these high-risk cases remains challenging. The authors aimed to develop a bedside prediction nomogram using routine ultrasound parameters.

## Material and Methods

The authors retrospectively analyzed 484 consecutive ultrasound-confirmed SBI cases (January 2020-December 2024). Inclusion criteria was isolated SBI, age 0–18 y old and complete data. Exclusion criteria was postoperative, ileocolic, pathological lead point, or missing data. Irreducible SBI was defined as failure of spontaneous reduction within 24 h or surgical requirement. The study was approved by the Ethics Committee of the First People’s Hospital of Lianyungang (Approval No.: KY-20250813002-02), with informed consent obtained.

With reported irreducible SBI incidence of around 10% and intending to construct a 5-variable nomogram, the principle of events per variable >5 for moderate model stability required more than 25 events [6]. A total sample size of 250 was estimated to suffice for model building and internal validation with 1,000 Bootstrap iterations. Eleven candidate predictors were screened by Least Absolute Shrinkage and Selection Operator (LASSO) regression with 10-fold cross-validation. Five variables with non-zero coefficients entered multivariable logistic regression: bloody stool, intussusception diameter, length, bowel wall thickness, and Doppler flow grade (0 = abundant, 1 = scattered, 2 = absent) [7]. A nomogram was constructed and internally validated by 1,000 bootstrap resamples.

## Results

**Fig. 1:**
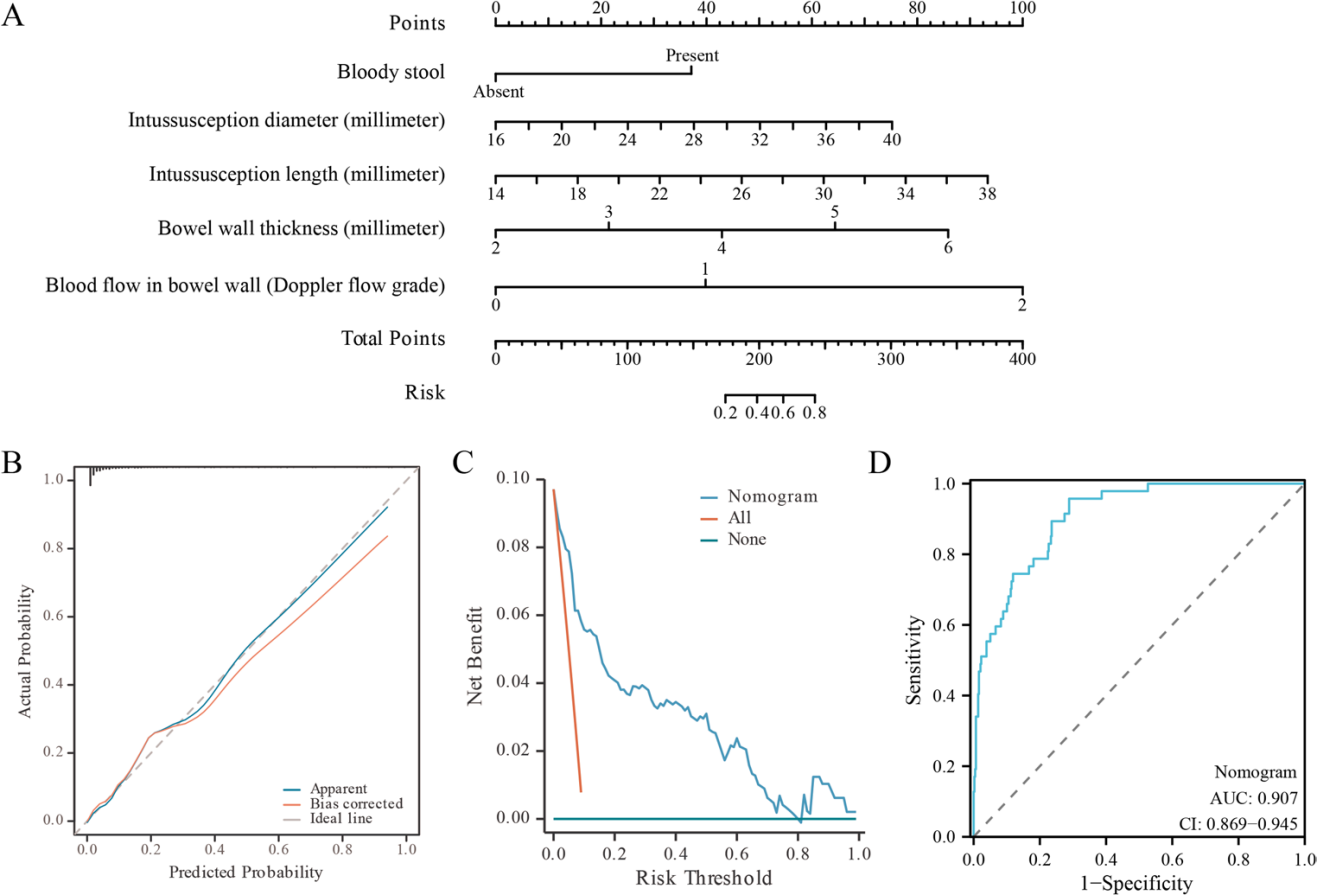
Nomogram predicting the risk of irreducible SBI. **(A)** Nomogram model, **(B)** Calibration curve, **(C)** Decision curve analysis and **(D)** ROC curve of the established predictive model

Baseline characteristics differed between transient (*n* = 437) and irreducible SBI (*n* = 47) in symptom duration, bloody stool, and all ultrasound parameters. The final model incorporated five routinely reported ultrasound parameters. The C-index was 0.907 (95% CI 0.869–0.945). The nomogram provides continuous risk estimation (total points 0-400 corresponding to probability 0.2–0.8). Higher scores indicate higher risk, enabling flexible clinical interpretation. The calibration curve showed excellent agreement between predicted and observed probabilities (Fig. 1).

## Discussion

This study developed a 5-variable nomogram for predicting irreducible SBI with a C-index of 0.907. The nomogram integrates bloody stool, intussusception diameter, length, bowel wall thickness, and Doppler flow grade into a single visual tool providing continuous risk estimation. All predictors are routinely available during standard ultrasound examinations, enabling 30-s bedside risk stratification without additional equipment. The excellent calibration and decision curve analysis support its clinical utility across diverse threshold probabilities. However, this single-center design with limited number of events, sparse data in the “absent flow” category, and potential measurement bias requires external validation before any clinical implementation.
